# A Paralytic’s Recovery

**Published:** 1889-02

**Authors:** 


### A PARALYTIC'S RECOVERY.

The following story of a remarkable recovery is vouched for by Ralph Wisner, keeper of the Ontario County poorhouse near the village of Canandaigua :

" Miss Pomeroy, about thirty-five years old, became an inmate of the poorhouse last winter after suffering a stroke of paralysis. Toward spring she had another stroke more severe than the first, and from that time until last evening she was unable to move a limb. She could communicate with her attendants only by whispers, and all hope of her recovery had been abandoned. Last evening she expressed a desire that one of the women of the house should come into her room and pray. Her wish was gratified, and after a short time the lady who responded was startled by a shriek from the stricken one, and when she saw the paralytic sitting up in bed she was so startled that she went out for assistance. When she returned the sick woman was partly out of bed. She continued to improve until she was able to dress and walk around the room. She believes that she has been restored to permanent health, and that it was through the efficacy of prayer. The physicians have no explanation to make regarding the astonishing cure.

